# An unusual cause of high anion gap metabolic acidosis: pyroglutamic acidosis

**DOI:** 10.1186/cc10755

**Published:** 2012-03-20

**Authors:** RJ Wardell, LA Burrows, K Myall, A Marsh

**Affiliations:** 1 Frenchay Hospital, Bristol, UK

## Introduction

Metabolic acidosis is a common acid-base disturbance in intensive care. A high anion gap indicates the presence of endogenous acids, which in critically ill patients are most commonly ketones, lactate and those accumulated in renal failure. However, excluding these causes means more rare forms of acid must be considered, including pyroglutamic acidosis. Pyroglutamic acidosis is caused by the accumulation of 5-oxoproline [1] due to the depletion of glutathione. This leads to loss of negative feedback and therefore the build-up of Y-glutamyl cysteine, which is converted to 5-oxoproline.

## Methods

During a 12-month period, three patients on our ICU with unexplained high anion gap metabolic acidosis had their urine screened for organic acids.

## Results

All had chronic methicillin-sensitive *Staphylococcus aureus *infections treated with long-term paracetamol and flucloxacillin. All cases presented to intensive care with reduced level of consciousness after several weeks of treatment. In each case, common causes of high anion gap metabolic acidosis were excluded and urine specimens contained grossly elevated levels of pyroglutamic acid. Flucloxacillin and paracetamol were stopped and *N*-acetylcysteine commenced, which led to resolution of the metabolic acidosis within 48 hours. All three patients made full recoveries. The first case has been previously described [2].

## Conclusion

Pyroglutamic acidosis is an uncommon condition, but should be considered in a high anion gap metabolic acidosis of unknown cause. The incidence in critical care may be more prevalent due to lack of screening currently. It is associated with sepsis, hepatic and renal dysfunction [3], and in patients who are receiving drugs such as paracetamol and flucloxacillin. If known precipitants are stopped, the condition can be rapidly reversed with full patient recovery.
